# Comparative Spatial Dynamics of Japanese Encephalitis and Acute Encephalitis Syndrome in Nepal

**DOI:** 10.1371/journal.pone.0066168

**Published:** 2013-07-22

**Authors:** Colin Robertson, Dhan Kumar Pant, Durga Datt Joshi, Minu Sharma, Meena Dahal, Craig Stephen

**Affiliations:** 1 Department of Geography & Environmental Studies, Wilfrid Laurier University, Waterloo, Ontario, Canada; 2 Institute of Medicine, Tribhuvan University, Kathmandu, Nepal; 3 National Zoonoses and Food Hygiene Research Centre, Kathmandu, Nepal; 4 Centre for Coastal Health, Nanaimo, British Columbia, Canada; Kenya Medical Research Institute - Wellcome Trust Research Programme, Kenya

## Abstract

Japanese Encephalitis (JE) is a vector-borne disease of major importance in Asia. Recent increases in cases have spawned the development of more stringent JE surveillance. Due to the difficulty of making a clinical diagnosis, increased tracking of common symptoms associated with JE—generally classified as the umbrella term, acute encephalitis syndrome (AES) has been developed in many countries. In Nepal, there is some debate as to what AES cases are, and how JE risk factors relate to AES risk. Three parts of this analysis included investigating the temporal pattern of cases, examining the age and vaccination status patterns among AES surveillance data, and then focusing on spatial patterns of risk factors. AES and JE cases from 2007–2011 reported at a district level (n = 75) were examined in relation to landscape risk factors. Landscape pattern indices were used to quantify landscape patterns associated with JE risk. The relative spatial distribution of landscape risk factors were compared using geographically weighted regression. Pattern indices describing the amount of irrigated land edge density and the degree of landscape mixing for irrigated areas were positively associated with JE and AES, while fragmented forest measured by the number of forest patches were negatively associated with AES and JE. For both JE and AES, the local GWR models outperformed global models, indicating spatial heterogeneity in risks. Temporally, the patterns of JE and AES risk were almost identical; suggesting the relative higher caseload of AES compared to JE could provide a valuable early-warning signal for JE surveillance and reduce diagnostic testing costs. Overall, the landscape variables associated with a high degree of landscape mixing and small scale irrigated agriculture were positively linked to JE and AES risk, highlighting the importance of integrating land management policies, disease prevention strategies and promoting healthy sustainable livelihoods in both rural and urban-fringe developing areas.

## Introduction

Japanese encephalitis (JE) is the leading cause of viral encephalitis in Asia [1]. It is a mosquito-borne disease caused by a flavivirus that cycles between birds, pigs and people [2]. Its distribution has, in recent years expanded and is expected to spread more widely geographically with anticipated changes in climate and land use [3]. Although less than 1% of people infected with the JE virus develop clinical disease, approximately 20–30% of cases are fatal and 30–50% of survivors have long-term neurological sequelae [1]. The global incidence of JE is unknown due to varying surveillance efforts and capacity, but Campbell et al. [1] estimated its annual incidence in 24 JE-endemic countries to be 1.8/100,000; 5.4/100,000 for children 0–14 years of age. The high case fatality rate, high rate of severe long lasting neurological symptoms and the majority of deaths occurring in children make JE a major public health problem [4].

Acute encephalitis syndrome (AES) is characterized by acute onset of fever, a change in mental status, and/or new onset of seizures. In tropical counties, the annual incidence of AES has been estimated to be 6.3/100,000 and perhaps up to 10/100,000 for children [5]. JE is the major identified cause of AES in Asia [6]. AES serves as the World Health Organization's clinical case definition for JE and is used by clinicians to recognize suspect JE cases. AES is not, however, specific for JE. It can be associated with a variety of other pathogens including other viruses, bacterium and parasites that co-exist in JE endemic countries. It has been estimated that a quarter of AES cases in Nepal are due to JE and that AES cases that are caused by JE have a worse prognosis than AES caused by other pathogens [6].

Nepal is a landlocked mountainous country with a population of approximately 29 million in 2011. Positioned on the slopes of the Himalayan range between China in the north and India in the south, east, and west, Nepal's landscape is characterized by three landscape types representing an altitudinal gradient from Tibet to India including the Himalaya (mountain region), the Hill areas of the inner Terai valleys, and the plains of the outer Terai. Monsoon rains support intensive agriculture (e.g., rice growing) and animal production (cattle, poultry and ruminants) on the Terai plains. Rice paddies support the breeding of *Culex tritaeniorrhynchus*, the dominant vector of JE in Nepal. Outbreaks occur with the onset of monsoon rains in July and usually end in October in endemic Terai areas, while recent shifts of JE to hill-region areas and the Kathmandu valley exhibit markedly different temporal patterns [7].

JE was first confirmed in Nepal in 1978 in the Terai districts near the Indian border [8]. Due to the complexity of interactions between landscape, vectors, reservoirs, and hosts, the epidemiology of JE remains poorly understood in Nepal as well as in many other parts of Asia. Limitations and variability in surveillance systems, health care systems and diagnostic capacity in endemic areas may be leading to misclassification of JE cases and non-JE cases [1], [4] as well as creating errors in estimating AES distribution, incidence and impacts. Many estimates of JE incidence depend on hospital based studies [1] which may fail to capture cases with mild symptoms that do not interact with the healthcare system. Nepali citizens are additionally challenged due to difficulty in accessing health facilities, poor perception of government or public health centres, gender differences in use of health care resources and/or high levels of poverty which may prevent people from presenting for diagnosis [9]–[12]. Because self-referral was the most common route of presentation to hospitals for Nepali AES patients in 2006–08, these impediments to accessing health services may present a detection bias for AES and JE surveillance and/or epidemiological studies. Additional biases can arise due to the reliance on serological tests, such as enzyme-linked immunosorbent assay (ELISA), for diagnosis. A retrospective study in Nepal found that 82% of JE positive cases were based on IgM ELISA, with 79% being based on a single serum sample [6]. While IgM ELISA is an accepted standard for JE serological diagnosis, both IgG and IgM based ELISA can cross-react with other pathogens, such as Dengue virus and West Nile virus [13]–[15].

Infectious diseases, such as JE, are driven by ecological and social processes which result in heterogeneous distribution of disease risk. Strategic allocation of JE control resources require detailed knowledge of at-risk populations. This becomes more critical for low income countries such as Nepal where there may be insufficient resources or capacity to provide vaccination, mosquito control or education programs for all citizens. Spatial analysis can assist in forecasting where at-risk populations are geographically located, identify social and environmental risk factors and thus help with disease control planning. But, such analyses are limited in their ability to accurately classify the outcome of interest; in this case, the distribution and abundance of JE [16]. Impoinvil et al. (2011), for example, reported on associations between spatial heterogeneity of JE cases and environmental variables in Nepal, finding links between weather and land-use variables. That study used AES cases with a positive anti-JE IgM ELISA test in serum or cerebrospinal fluid, potentially missing cases that were not subject to diagnostic testing. Climate variables and land-use were similarly seen as geographic risk factors for viral encephalitis in Thailand, yet again, that study recognized the limitations created by inconsistent application of a rigourous inclusion criterion for case detection [17] . Given that; (i) access to diagnostic testing and reliance on hospitalized cases may exclude some JE cases from surveys or surveillance data; (ii) there are some overlaps in known risk factors for JE as well as some other diseases that may cross-react with JE on diagnostic testing and (iii) Rayamajhi et al. [6] found no marked differences in the season or age profiles of JE and non-JE cases among cases of viral AES presenting to hospital; we wondered if the geographic distribution and spatial risk factors for AES in Nepal would be sufficiently parallel to those for JE to allow AES to act as a syndromic surrogate for JE to inform public health planning.

Spatial analysis of syndromic surveillance has been used in other settings to detect disease clusters, track the spread of disease, identify spatial variability, and provide early warning for infectious and non-infectious diseases [18]–[21]. Syndromic surveillance has also been used to examine associations between environmental risk factors and disease outcomes [22] and for identifying high risk populations [23]. Our main research objective was to compare and contrast spatial risk factors of AES and JE in Nepal in order to explore the potential for AES syndromic surveillance to inform public health decision making for JE prevention and control.

## Methods

Geographical patterns can provide important clues about disease etiology [24]. The objective of this analysis was to examine whether the cases reported as AES were similar in their geographic pattern to cases identified as JE. It is hypothesized that a certain proportion of AES and JE cases are due to the same etiological processes. We examined relationships between geographic risk patterns and risk relative to underlying landscape variables related to etiologic processes specific to known JE epidemiology. Finally, we compared risk factors and geographical patterns in risk factors to further elucidate the epidemiological relationship between JE and AES in Nepal.

### Data

AES and JE surveillance data were obtained from the AES Surveillance System as part of the World Health Organization (WHO) Programme for Immunization Preventable Diseases (IPD) which is responsible for many types of disease surveillance throughout Nepal. Cases are obtained from reporting district health centres (578 for all of Nepal) and tested for JE using the anti-JE IgM ELISA(X CYTON commercial Kits which has sensitivity 77.8% and specificity 93.3%) test. Cases testing positive for JE were extracted and classified as JE, while cases testing negative were denoted as AES. A small subset of cases was not tested or the test results were inconclusive, we classified these cases as unknown viral encephalopathy (UVE).

**Figure 1:**
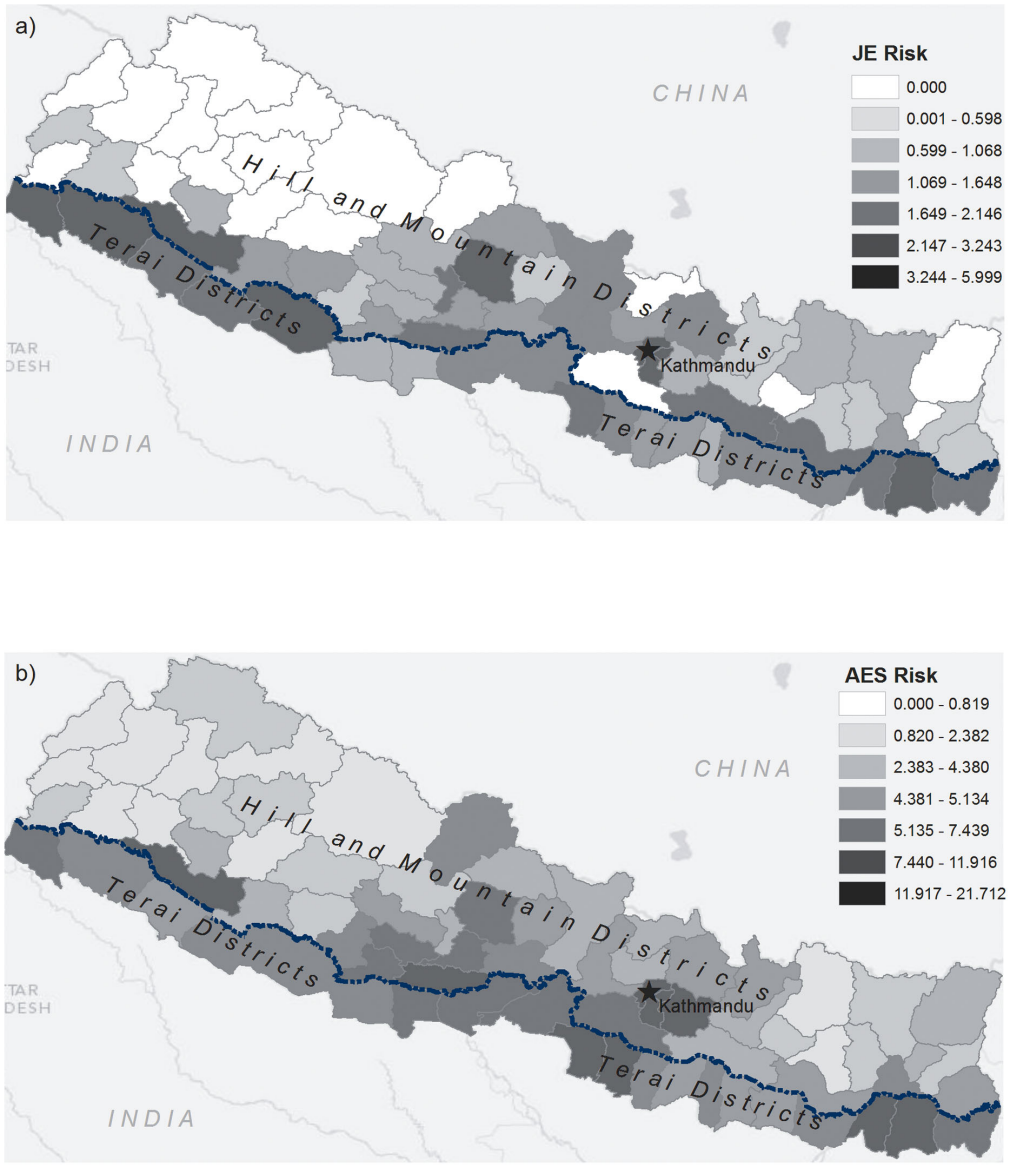
Maximum standardized incidence ratios over the study period for a) Japanese Encephalitis (JE) and b) Acute Encephalitis Syndrome (AES).

Disease data were integrated with population data obtained from the Nepalese Department of Health Services for each of the study years (2007–2011) to compute year-specific incidences for each district. While geographic studies of disease risk often deal with unstable risk estimates resultant from low population areas via Bayesian shrinkage estimation [25], this was not done here because the empirical risk distribution for both JE and AES is not related to sample size and discrete discontinuities in risk may in fact, be a real feature of JE epidemiology. Further, the large size and relatively low number (n = 75) of districts negated any procedures which could potentially oversmooth and misrepresent the true risk distribution [26]. The maximum annual incidence rates for the study area and years are presented in Figure 1. Maximum annual incidence was used as a measure of risk as our interest was in identifying outbreak prone locations and visual analysis. For Poisson regression-based risk modelling, the 2009 population estimates were used as the denominator data (i.e., offset variable).

Landcover data was obtained from the South Asia edition of the Global Land Cover 2000 Dataset (GLC) which is a global mosaic of SPOT-4 data produced by the Joint Research Centre of the European Commission [27]. The global mosaic is an aggregation of regionally-optimized landcover classifications with a harmonized globally integrated legend. The South Asia GLC data included all of Nepal which contained 27 of the 47 landcover classes defined for South Asia. The South Asia classification is based on multi-temporal SPOT Vegetation data whereby temporal profiles were matched to phenological variation in vegetation types to improve classification accuracy [28]. A validation study of the South Asia classification map found forest and cropland classes to be 78% and 74% accurate relative to high-resolution classifications [29]. The landcover dataset was clipped to the boundaries of Nepal and used to characterize landcover within each of the districts.

### Landscape Pattern Indices

Landscape pattern indices (LPIs) are measures of landscape structure that typically characterize one of two fundamental properties of spatial pattern: configuration, the spatial arrangement of landscape patches, and composition, the variance and abundance of patches within a landscape. LPIs are used to summarize properties of spatial pattern, usually with a single value for a given landcover map. Measures of configuration, such as patch size distribution, habitat connectivity [30] and the density of edges [31] offer different ways of measuring how the landcover types in a landscape are arranged. Compositional metrics on the other hand, such as the number of patch types, relative abundance of each patch type, and composite measures [32] relate to the overall abundance of specific landcover classes in the landscape.

LPIs have been used to reveal how landscape can function to impact emerging infectious diseases. For example, Langlois et al. examined landscape pattern indices in relation to hantavirus in deer mice, finding that both forest composition (i.e., deer mice habitat) and configuration (i.e., fragmentation) were related to hantavirus geographical distribution [33]. LPIs may have special utility for vector-borne diseases due the environmental sensitivity of vector habitat locations which can vary greatly over small spatial scales and be important causal factors in human infection risk. Through simulation, Smith et al. [34] showed that the biting rate of infectious mosquitoes declines with distance from larval habitat, while the percentage of mosquitoes that are infectious increases with distance due to shifts in age distributions with dispersal from larval breeding areas. Thus, when humans and mosquito populations are distributed heterogeneously, the density of infected humans was found to peak at a distance far from the source of the mosquito populations. These results imply that it is not the mere presence of mosquito habitat that impacts the risk of human infection, but also the spatial arrangement of habitat patches in relation to human population density.

While conceptually, the notions of composition and configuration describe two distinct aspects of spatial pattern, measures of these properties are inherently correlated [35], and there is need to determine the extent to which changes in configuration are due only to compositional change [36]. LPIs also do not map uniquely to spatial patterns, leading to the possibility of perceptually (and statistically) different landscapes having identical LPI values [37]. Finally, landscape patterns are scale dependent [38] and require scale-specific interpretations of underlying mechanisms when used in a quantitative analysis.

Correlation among indices is usually handled through correlation tests or ordination techniques [35]. Non-unique mapping between LPI values and landscape patterns applies primarily to assessing landscape change and has been addressed by simulating landscapes with known levels of composition and configuration [39]. Scale issues are usually accommodated by repeating analysis at multiple scales, framing LPIs within a hierarchy of landscape patterns and ecological processes [40]–[41]. In all research contexts, careful selection of LPIs based on hypothesized effects on an ecological process is a key requirement [42]. In spatial epidemiological studies, LPIs are best applied in a large-scale exploratory studies to provide evidence for existing hypotheses of disease mechanisms or to suggest new hypotheses.

We selected LPIs that characterize the both spatial configuration and composition of landcover classes relevant for JE epidemiology. The transmission cycle of JE in Nepal is suspected to include humans exposed to the JE virus via mosquito bites. Mosquitoes also transmit the virus to pigs, which serve as an amplifying reservoir. The source of the virus is generally thought to be migratory birds in most of Asia. The role of landscape can factor into any of these epidemiological links. For example, Ferraz et al. [43] showed that increasing fragmentation in Brazilian forests decreased avian biodiversity, which could have important effects given the complex relationships between biodiversity and emerging infectious disease ecology [44]–[45]. Landscape pattern may also impact mosquito populations, Yasouka and Levins [46] reported significant effects of deforestation and agricultural development of anopheline mosquito populations, noting for example the fourfold increase in malaria incidence in Afghanistan reportedly due to irrigation-based agricultural development [47]–[48].

**Figure 2:**
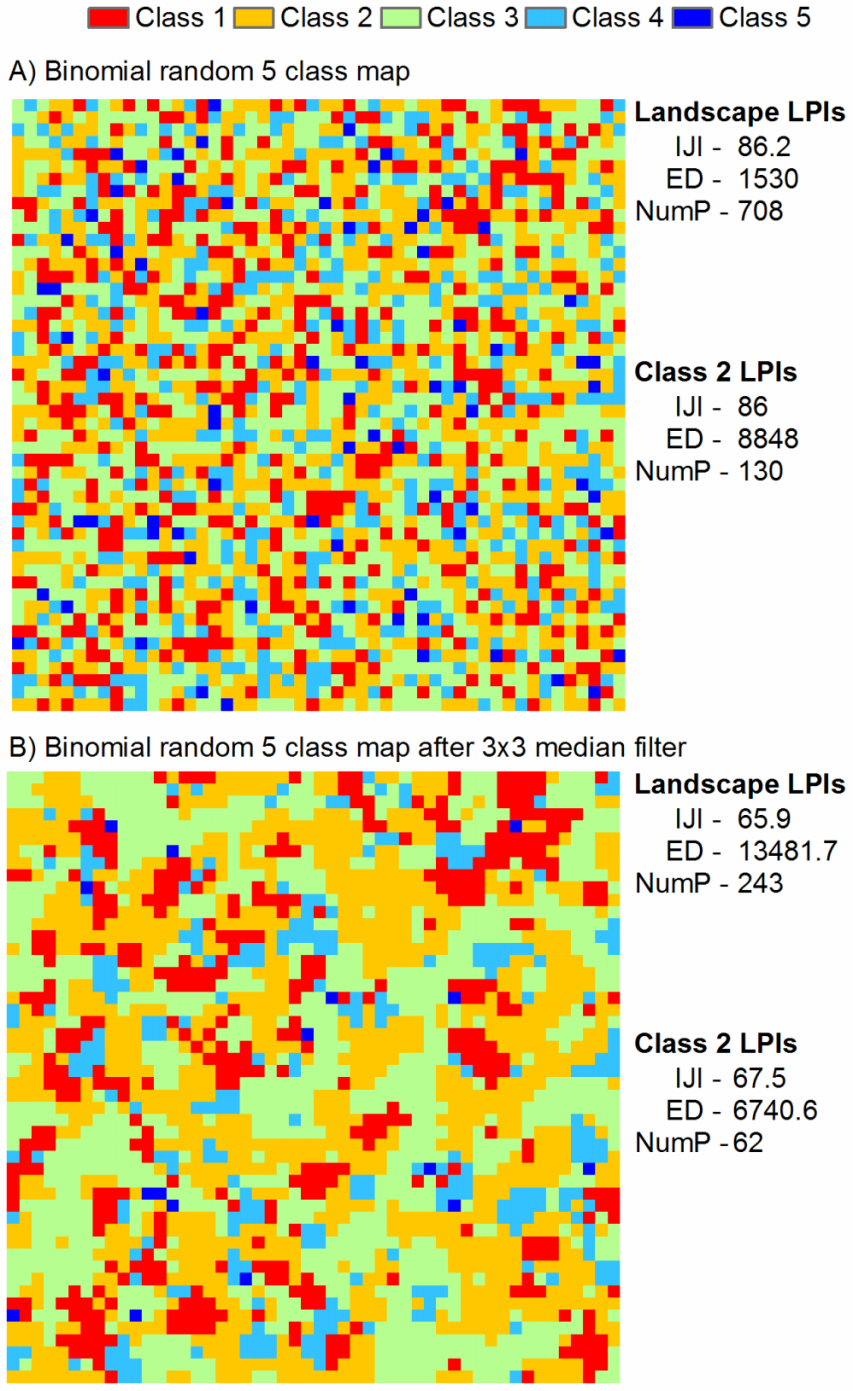
Landscape pattern indices (LPIs) used to investigate relationships between landcover and disease risk. The interspersion juxtaposition index (IJI) measures the relative spatial mixing of a landcover class in the study area. Edge density (ED) measures the amount of edge relative to the total area (class and landscape), while the number of patches (NumP) reports the total and class level number of contiguous landcover classes. Class 2 was chosen to illustrate class level LPIs in the random map drawn from a Binomial distribution (a) and the result of a median filter on the same map (b) which maintained the relative proportion of class 2 (∼32%).

Landscape types extracted from the landcover inventory were based on the GLC 2000 Legend specific for South Asia. Table 1 outlines the landcover classes and aggregations used for this analysis. The most important landcover types thought to impact JE risk were forest (i.e., bird habitat), irrigated land (i.e., mosquito breeding sites and exposure sites), and grasslands. These classes were used as input to LPIs (Table 1). To quantify the relative abundance of each patch type within each landscape, the total area of each relevant landcover type was used. An illustration of each of the LPIs is outline in Figure 2. As can be seen for Class 2, the number of patches (NumP), the interspersion index (IJI) and the edge density metric (ED) are all higher for a randomly drawn map than the clustered (and much more realistic) map. These measures therefore provide some indication of the relative mix of the different landcover types and may have utility as proxy risk factors in large-area studies.

**Table 1:** Landscape pattern indices used to quantify landscape patterns in Nepal based on Global Land Cover 2000 classification.

Metric	Definition	Rationale
Grassland Area (G_sum)	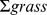	Bird habitat, associations found in previous studies of spatial JE epidemiology
Irrigated Agriculture Area (I_sum)	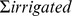	Mosquito habitat associated with standing water and rice paddy agriculture
Forest Area (F_sum)	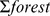	Bird habitat associated with long-range virus movement.
Irrigated Agriculture Interspersion Juxtaposition Index (I_IJI)	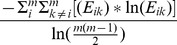	A measure of how well mixed the land cover configurations are in the District. If irrigated agriculture areas are the source, a mixed distribution may increase exposure to infected mosquito populations.
Number of Forest Patches (F_NP)	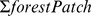	A measure of forest fragmentation that may influence mixing of bird and mosquito populations.
Average Edge Density of Irrigated Agriculture (I_ED)	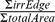	A measure of the amount of edge of irrigated agriculture relative to the total area of the district

All indices are computed at the district level.

Each district was used as the unit of analysis for computing LPIs as these were the scale at which disease data were obtained. Distributions of LPIs were compared against JE and AES risk using descriptive statistics and graphs. The Patch Analyst Extension [49] and FragStats [50] were used to compute LPIs.

### Geographically-weighted regression

Geographically-weighted regression (GWR) is a local regression method that generates *N* spatially localized regressions between a dependent variable *y* and a vector of covariate variables observed over the same geographic units [51]. The basic GWR models is specified as
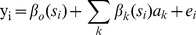
(1)where the ordinary global regression model is rewritten with local (*s_i_ = x_i_,y_i_*) estimates for the intercept β_0_ and slopes β_k_. In our case, because we are working with count data, we used the generalized linear model GWR with a Poisson link function. This generalizes the ordinary GWR model to the generalize linear modelling framework, making the local model as follows
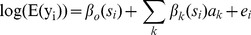
(2)This model provides flexibility to see how relationships between covariates and the dependent variables vary across space. The GWR model is calibrated with a distance decay model such that points in the neighbourhood of the estimation location *i* closer to location *i* are weighted more than points further away from *i*. The form of the distance decay weighting function varies, but in this analysis a Gaussian formulation was used as
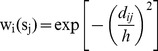
(3)where *d_ij_* is the distance between centroids of the district *i* and district *j*, and *h* is the bandwidth. Selection of appropriate weighting function can be considered a model selection issue and is generally considered less important than the definition used for spatial neighbours [51]. The neighbourhood definition (i.e., what points are included in the local regression) represents a trade-off between lowering standard errors of local coefficient estimates with increasing local sample size, and increasing bias into estimates by incorporating points that are ‘further away’ and potentially unrelated to the estimating location. While automated neighbourhood definition methods are available using metrics such as the AICc and cross validation, in this analysis we focused specifically on local neighbourhoods – as our intent was descriptive rather than explanatory (i.e., we wanted to compare local epidemiological risk factors for JE and AES rather than fit the best model possible). As such, we used a k-nearest neighbours definition with K = 15, determined after exploratory analysis.

The purpose of GWR is to reveal underlying spatial non-stationarity in relationships identified in a regression model, and normally occurs following a global regression model and inspection of model residuals. Here, we first specify a global regression model where the risk of JE in district *i* is related to the coefficients for the amount of area devoted to each of the landcover classes and the relevant LPIs (Table 1).

Finally, as our objective was to identify differences in pattern of JE and AES risk in Nepal, basic epidemiology of the time series of cases is explored. We computed time series for each of the three types of disease as well as other variables that may shed light onto the etiology of the AES cases, including the proportion of cases presenting that had been vaccinated with JE vaccine, and the proportion of cases reported by individuals under the age of 16. Through joint analysis of the temporal, spatial, and landscape distributions of JE and AES cases, we aimed to uncover new insights into their respective epidemiology.

## Results

The total number of cases reported to the AES Surveillance System in Nepal peaked in 2008 with 1988 total cases, while the lowest number of (1216) cases reported was in 2011 (Table 2). The greatest decline was in laboratory confirmed JE cases, while AES declined also in 2011. The trend in lab-confirmed JE reflects the mass vaccination campaign initiated in Nepal in 2005, which aimed to vaccinate all children under the age of 15, with initial efforts focused in rural areas, and in later years around the Kathmandu valley. The spatial distribution of JE and AES risk in Figure 1 illustrates two common patterns of risk. Firstly, rural areas in the northern and southern Terai districts have a high relative risk profile. This fits with expected patterns of risk for confirmed JE, although not necessarily for AES. Secondly, high risk is also noted in the Kathmandu Valley districts, as has been previously reported. Importantly, the pattern holds for both AES and JE risk. Very few cases are observed in the remote western districts. In terms of risk magnitude, incidence varies from zero to almost 6 for JE, and up to over 21 cases per 100,000 for AES.

**Table 2:** Descriptive statistics of Japanese Encephalitis (JE), Acute Encephalitis Syndrome (AES) and Unknown Viral Encephalopathy (UVE) in Nepal, 2007–2011.

	2007	2008	2009	2010	2011
JE	442	339	147	197	129
AES	1142	1548	1274	1305	966
UVE	73	101	97	112	121
Totals	1657	1988	1518	1614	1216

**Figure 3:**
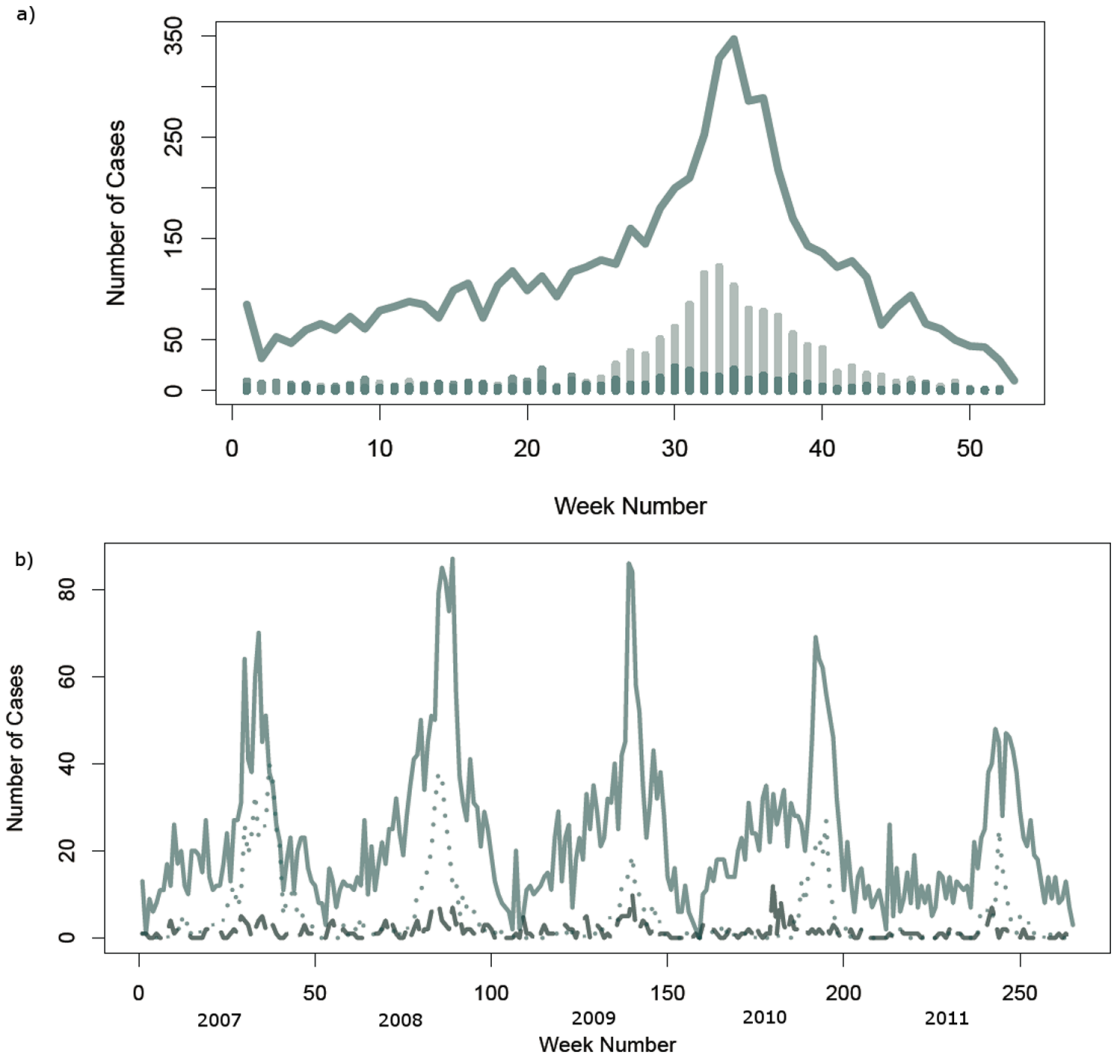
Temporal patterns in Japanese Encephalitis (JE), Acute Encephalitis Syndrome (AES) and unknown viral encephalopathy (UVE) in Nepal, 2007–2011. a) Seasonal distribution of AES (line), JE (light bars) and UVE (dark bars) cases over the study period. b) Time series of AES (solid), JE (light dots) and UVE (dark dash) cases over the study period.

**Figure 4:**
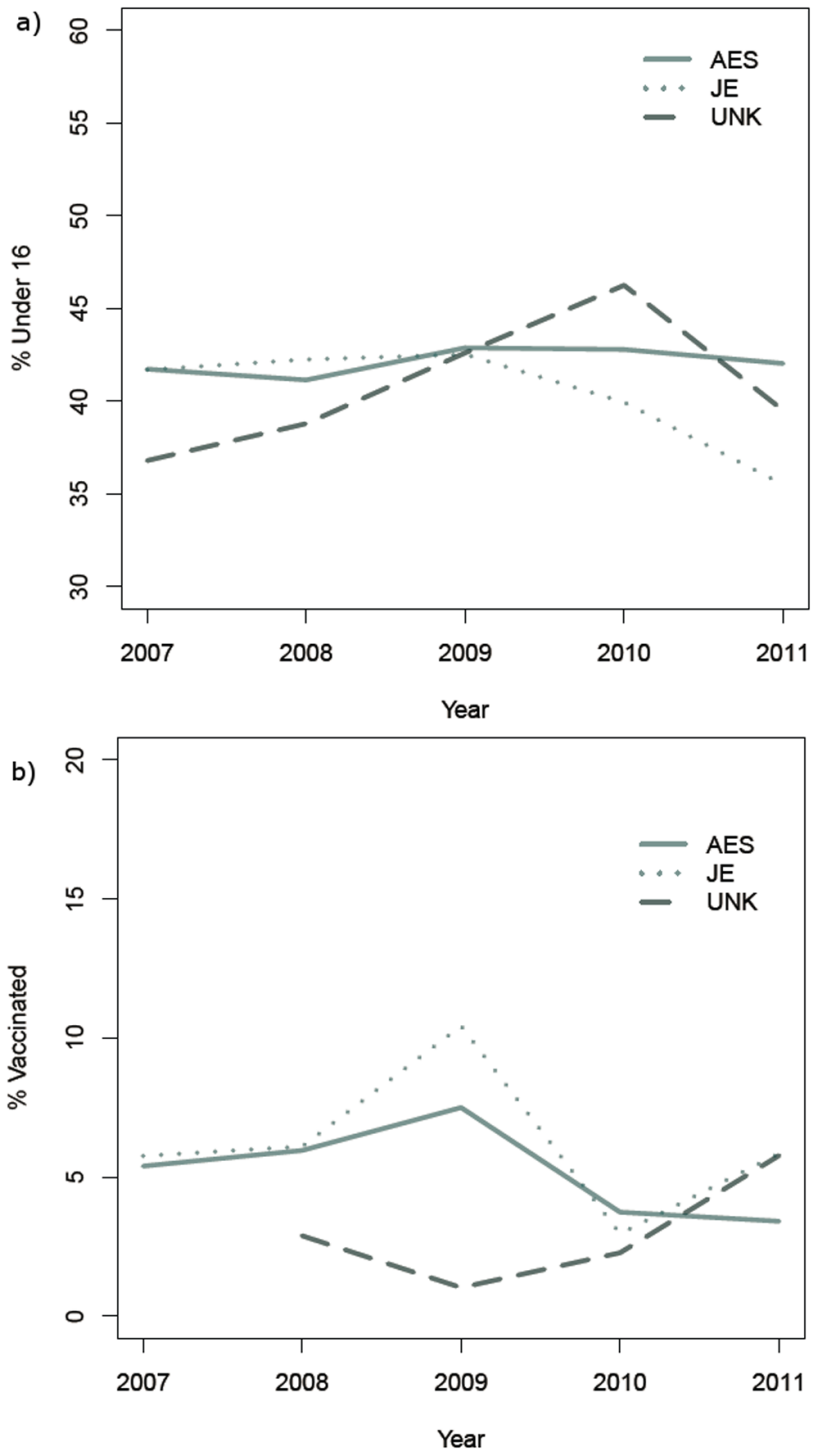
Annual trends in a) the proportion of cases under the age of 16 and b) the proportion of cases vaccinated in Nepal, 2007–2011. (JE - Japanese Encephalitis, Acute Encephalitis Syndrome (AES), unknown viral encephalopathy UVE).

The seasonal dynamics associated with JE is evident for AES also (Figure 3a), with identical peaks in cases in late summer and early fall, while for UVE there is a relatively even distribution throughout the year. Over the course of the study period, the seasonality of AES dampened in 2011 (Figure 3b). Whether this pattern can be explained by the vaccination campaign is unclear. The percentage of cases in individuals under the age of 16 is presented in Figure 4a for JE, AES and UVE and vaccinations over time are presented in Figure 4b. Each of these represents important clues as to the interaction between JE and AES epidemiology. If for example, vaccination status differed significantly for AES and JE, we would have clear evidence for both the efficacy of the vaccination campaign, and the existence of non-JE aetiologies associated with AES. However with vaccinations barely reaching more than 10% of cases, it is not possible to use this as a discriminator between JE and AES. Additionally, the proportions of patients under the age of 16 were fairly consistent over the study period, composing between 35–45% of the total caseload for JE, AES and UVE (Figure 4a).The similarity (and high percentage) of cases under the age of 16 for all disease types is an analogous finding to the similarity in temporal pattern and geographical risk factors – that is, the exposures for these diseases are constant across age, and likely due to a common set of exposure mechanisms. Neither age distribution nor vaccination status appear to discriminate among the disease classifications examined in this study.

The global regression model results relating JE and AES to landscape variables are outlined in Table 3 (UVE was not including in modelling due to the relatively low number of cases). For both models, the composition variables (grassland area, forested area, irrigated area) had no significant relationships with either JE or AES and were excluded from the final model results reported here. The JE Poisson regression model was determined to be significant (R^2^ = 0.31, AICc = 649.856) with irrigated area edge density and irrigated area land mix having significant positive relationships with JE incidence, and number of forest patches a significant negative relationship (α = 0.05). The global regression model relating AES to landscape variables was also significant (R^2^ = 0.38, AICc = 2075.794) with all three variables significant (α = 0.05) predictors of AES incidence.

**Table 3:** Global regression model results for a) Japanese Encephalitis (JE) and b) Acute Encephalitis Syndrome (AES).

a) JE Global Poisson Regression Model Results		
Variable	Coefficient Estimate	Std. Error
Intercept	−10.260	0.038
Irrigated Edge Density	0.239	0.035
Number of Forest Patches	−0.259	0.048
Irrigated Land Mix	0.230	0.024

b) AES Global Poisson Regression Model Results		
Intercept	−8.699	0.018
Irrigated Edge Density	0.137	0.137
Number of Forest Patches	−0.442	0.231
Irrigated Land Mix	0.255	0.011

Note that coefficient estimates are reported in log terms and the independent variables were z-transformed before entering into the regression model.

**Figure 5:**
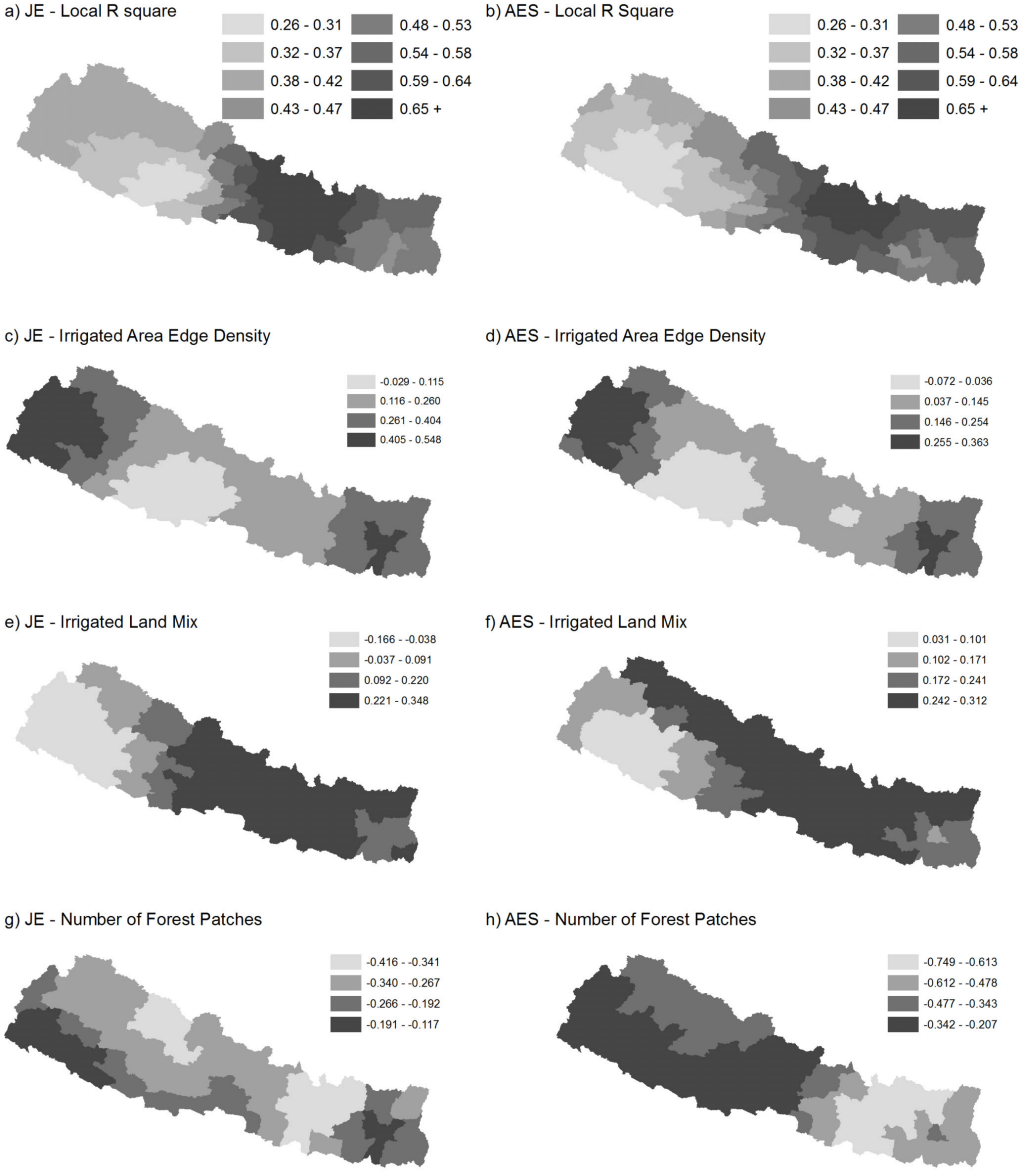
Maps of geographically weighted regression analysis for Japanese Encephalitis (JE) and Acute Encephalitis Syndrome (AES) and relationships with selected landscape pattern indices.

The results of the geographically weighted regression analysis are presented in Figure 5, which gives the spatial distribution of local R^2^ values for JE and AES (a–b), as well as the coefficient maps for the landscape variables (c–h). Generally, the JE model (R^2^ = 0.584, AICc = 420.215) and the AES model (R^2^ = 0.549, AICc = 1535.086), had a similar spatial distribution of local R^2^ values, with the highest values located around the Kathmandu valley area. The pattern in coefficient estimates (represented with 4 equal interval classes) were similar as well. The highest coefficient estimate values were found for irrigated land mix. For irrigated area edge density, the variable with the second largest coefficient magnitude in the models, the spatial distributions highly similar between AES and JE. For high risk areas in the Terai, the edge density variable had a high positive effect on risk of both JE and AES when compared to areas in and around the Kathmandu Valley. The number of forest patches variables had a negative relationship with both JE and AES risk, however the patterns differed between JE and AES. Overall, the negative effect was of a higher magnitude and more spatially uniform for AES than for JE. A comparison of the global models and the GWR models for both JE and AES based on the AIC revealed that the local model was the better model for both JE (Δ AICc = 299.641 – 35.3%) and AES (Δ AICc = 531.708 – 25.7%).

## Discussion

The combined burden of AES, JE, and UVF represent a significant and continuing public health issue for Nepal and other Asian countries. While confirmed laboratory cases of JE represent only 15.6% of total cases reported to the AES Surveillance System during the study period, the analysis presented here demonstrates that AES cases are a useful syndromic surveillance signal for managing JE as public health issue. Temporally, the pattern of cases of JE and AES is very similar (Figure 3a) and likely due to the higher case volume, the rise in cases associated with summer high season in AES precedes that of JE, suggesting a significant early-warning value for public health planning (Figure 3b). Random variation in low numbers of JE cases does not reveal the size of the seasonal caseload, while examining the AES time series, the case load early in the year consistently predicts the size of the peak season case load. Where public health resources are scarce, this analysis presents cursory evidence for the syndromic value of AES for timely early-warning to supplement more costly laboratory testing.

Interestingly, the amount of land cover for each landcover type (grassland, irrigated areas, forest) was not related to either JE or AES risk. This is surprising in that JE is typically regarded as a disease endemic to rural rice paddy areas in Asia. Recent trends towards establishment of JE in the Kathmandu Valley and mountain districts [52]–[53] were confirmed in this analysis (Figure 1), and thus may have masked the expected relationship with the amount of irrigated area (i.e., rice paddy agriculture). However, the density of irrigated area edge was significantly associated with both AES and JE risk, representing both mosquito breeding habitat and potential exposure surface area. This finding highlights the importance of spatial configuration measures in spatial epidemiological analyses. Further, the relationship was not uniform, with moderate positive relationship between AES/JE risk and edge density in the northern and southern Terai districts (Figure 5c/d).

To our knowledge, this is the first association found at the landscape level between rice paddy spatial configuration and disease risk in Nepal. In a recent analysis [16], the percentage of irrigated land was found to have a slight positive relationship with lab-confirmed JE in 2007. This effect found was small (β = 0.028) and was not significant in 2008 (the last year of the study's data). It is likely that the effect in [16] represent rural Terai endemic JE, whereas the association identified in this analysis also measures risk in the Kathmandu Valley districts. The coefficient map in Figure 5c supports the homogeneity of the effect in the northern and southern Terai. From a land management perspective, the results indicate heightened risk of both AES and JE where small scale irrigation agriculture mixes with other land cover types (Figure 5e/f). This was the anticipated finding, as greater mixing and amounts of edge represent greater opportunities for exposure to disease vectors, it also represents a challenge in the promotion of healthy landscapes. For example, the location of settlements proximal to the small-scale rice paddy area may both support food security and sustainability, but also incur greater disease burden due to vector-borne disease risk. Public health planning and disease control activities in Nepal should consider the role of land use mix in promoting healthy land management planning rather than purely land use amount. For example, irrigated agriculture in the Terai versus steppe rice agriculture in hill regions may differ significantly in terms of area, but confer the same risk of vector borne disease exposure. This spatial configuration of irrigated areas may also be a proxy for virus mixing between egret, pig, and mosquito populations, a process not well understood but often cited as a key driving process in the emergence of new zoonoses in developing regions. The negative relationship identified between forest fragmentation and JE/AES is difficult to understand, other than that it is potentially not related to the virus reservoirs as was suspected. More thorough investigation of wild and domestic avian host reservoirs are needed to determine this conclusively.

There are important caveats to the spatial analysis of JE and AES risk at the district scale which should be highlighted and may impact our reported results. Firstly, the large size and relatively low number of districts limits the specificity of risk factor relationships which could be identified. Secondly, the case data is subject to significant time delays and variation in reporting/surveillance effort. The AES surveillance system utilizes 578 sentinel sites for data collection, however the accessibility of these sites is not uniform, and variation in case detection may impact the patterns reported here. From a methodological perspective, it is important to reiterate the descriptive and comparative purpose of the models presented here. Our ultimate objective was to compare the patterns of JE and AES risk and landscape-oriented risk factors rather than develop the best fitting model for predictive and/or explanatory purposes. GWR is an exploratory technique which highlights non-stationarity in estimated linear relationships and is not suited to rigourous estimation of risk factor effect sizes. Future analysis should further explore the relationships between spatial configuration of irrigated lands and JE and AES risk using more robust techniques – for example, incorporating a joint-risk model at a more local spatial scale [54].

The spatial, temporal, and landcover configuration distributions of JE and AES were explored in this study. Overall, we found a high similarity in pattern between JE and AES across all three dimensions of comparison.
